# Synthesis of a Bimetallic-Doped Phytate-Melamine Composite as an Efficient Additive for Epoxy Resins with High Fire Safety

**DOI:** 10.3390/polym16243586

**Published:** 2024-12-21

**Authors:** Shunxiang Wang, Jianfeng Huang, An Wei, Yulian Chen, Xulan Lu, Yongjin Zou, Fen Xu, Lixian Sun, Yunhao Lu, Cuili Xiang

**Affiliations:** 1 College of Materials Science and Engineering, Guilin University of Electronic Technology, Guilin 541004, China; 2 School of Materials Science and Engineering, Zhejiang University, Hangzhou 310027, China

**Keywords:** melamine, phytic acid, bimetallic, flame retardancy

## Abstract

The issue of hazardous smoke and toxic gases released from epoxy resins (EP), which often causes casualties in real fires, has limited its application. Therefore, we have developed a novel flame retardant based on a bimetallic-doped phytate-melamine (BPM) structure with Zn^2+^ and Fe^2+^ ions incorporated into the polymer matrix using a straightforward solution-based synthetic method. The combustion performance of the composite was evaluated using a cone calorimeter test, which showed that the peak heat release, total heat release, and total smoke production were reduced by 50%, 31.7%, and 29.2%, respectively, compared to those of EP. Additionally, the fire growth index was noticeably reduced by 60% owing to the synergistic catalytic effect of the bimetallic ions, and the high nitrogen and phosphorus content of the additives. Overall, this study provides new insights into the application of bimetallic doping for flame retardant polymer composites.

## 1. Introduction

Epoxy resins (EP) are widely applied for various purposes, such as coatings, electronic encapsulation materials, and advanced composite materials, owing to their superior comprehensive performance. However, their flammability and production of harmful smoke particles during combustion can cause secondary harm to human health and damage to the environment. To meet fire safety requirements, especially in the field of coatings, the flame retardancy of EPs has become a focus of research, which can be significantly improved by physical or chemical methods. Physical methods include the addition of inorganic fillers, such as aluminum hydroxide [1] and magnesium hydroxide [2], which decompose at high temperatures and absorb heat while releasing water, thereby lowering the temperature of the material and inhibiting combustion. Chemical methods involve the introduction of flame retardant elements, such as halogens or phosphorus compounds, into the molecular structure of the epoxy resin, which form a stable carbon layer during combustion, thus insulating the material from oxygen and heat and effectively slowing down the combustion process. For instance, Klingler et al. applied an epoxy resin composite coating to the surface of wood, and showed that the peak heat release rate (PHRR) and the total smoke production (TSP) were reduced by 75% and 72.5%, respectively [3]. Ravikumar et al. demonstrated that the incorporation of Py(SrO-CuO) nanocomposites into intumescent flame retardant coatings increased the LOI value to 28.5% [4].

Phytic acid (PA) is a green, non-toxic, and sustainable bio-based phosphorus-containing organic acid that possesses six phosphorus groups and phosphorus content as high as 28 wt.% per molecule [5]. These phosphorus groups endow PA with the ability to bind carbohydrates and fabrics, rendering it a highly promising flame retardant derived from biomass [6]. It has thus been widely used in the synthesis of EP flame retardant coatings [7]. The phosphoric acid groups of PA undergo dehydration to form coke at high temperatures, thereby delaying the heat transfer and preventing the release of flammable gases during combustion. Its flame retardant efficiency has been confirmed in many materials, including polymers [8] and cotton fabrics [9,10]. However, the use of PA, alone or in combination, can lead to partial degradation of the substrate owing to its strong acidic properties. This results in damage to the structure of the matrix material, which reduces the mechanical properties of the matrix. It is therefore important to limit the negative effects of PA when used on its own while ensuring flame retardancy. Intumescent flame retardants (IFRs) containing phytic acid as the acid source and melamine as the nitrogen source have attracted considerable attention owing to their high efficiencies [11,12]. For example, melamine phytate (MPA) was prepared as an IFR for polypropylene (PP) [13] and EPs [14].

Metal-containing smoke suppression agents are widely used to reduce smoke emissions by improving flame retardancy during the condensation phase [15]. Phytic acid can form insoluble complexes (phytates) with metal ions, thus building an effective flame retardant barrier inside wood and enhancing its fire resistance [16]. Monometallic doping is widely used owing to its low cost and simplicity. By incorporating a solution of phytic acid, magnesium ions, and tannic acid into polyvinyl chloride, Meng et al. achieved a UL-94 rating of V-0 and LOI as high as 30.3% [17]. However, the flame retardant effects of individual metal ions are limited. Therefore, low-cost multi-metal synergies can be leveraged to fully utilize the strengths of different metallic elements, thus complementing the advantages imparted by each metal to achieve better performance. It is important to note that melamine can cause health hazards, including nephrotoxicity, when ingested in large amounts. Zinc and iron, although essential nutrients, can also be toxic when ingested in excess. Exposure risks may occur during synthesis, processing or degradation, so proper handling protocols, protective equipment and safety guidelines during production and application are essential to mitigate potential hazards.

Therefore, BPM were synthesized using the water bath method using Zn^2+^ and Fe^2+^, which are highly abundant, inexpensive, and in-line with the requirements for sustainable development. In particular, Zn^2+^ exhibits high thermal stability and can form a stable charcoal layer during combustion, thus effectively isolating oxygen and heat transfer. Similarly, Fe^2+^ can catalyze the formation of a charcoal layer during the flaming process because of its high redox activity, which can further improve the flame retardancy efficiency. Moreover, the phosphorus and nitrogen elements in PA and MEL promote the densification of the carbon layer during pyrolysis, which enhances the flame retardant barrier effect. Finally, the EP with BPM flame retardant was coated onto the surface of wood to test performance in practical applications. From the experimental results, it can be seen that BPM improves the thermal stability, char formation, and smoke suppression of the EP, which limits the hazards to humans and the environment in event of fire. This study provides new insights into the flame retardant properties of bimetallic doped PM-modified EP, which will lay the foundation for the development of low-cost, high-efficiency, and environmentally friendly flame retardants.

## 2. Experimental Section

### 2.1. Materials

EP (E-44, CAS number: 61788-97-4) was obtained from Qingtian Chemical Co., Ltd. (Jinan, China). Melamine (MEL, CAS number: 108-78-1), ferrous acetate (CAS number: 3094-87-9), zinc hydroxide (CAS number: 20427-58-1), ethylenediamine (EDA, CAS number: 107-15-3), and phytic acid (PA, 70 wt.% in water, CAS number: 83-86-3) were purchased from Aladdin (Shanghai, China), and used as received. Distilled water (DI) was used in all experiments.

### 2.2. Synthesis of BPM

**Figure 1:**
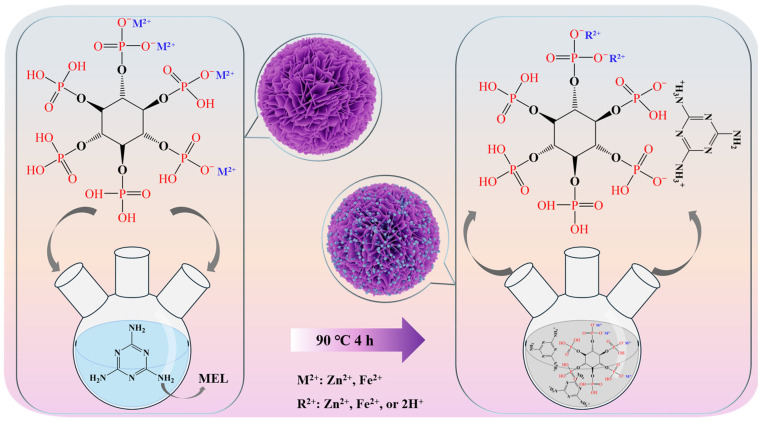
Fabrication process of the BPM flame retardant.

The process for preparing the flame retardant is shown in Figure 1. Firstly, 6 mmol of PA was dispersed in 60 mL of DI, to which 4.5 mmol of ferrous acetate and 4.5 mmol of zinc hydroxide were added to obtain the Zn-Fe bimetallic phytate solution (BP). 24 mmol of MEL was then dissolved completely in ultrapure water at 90 °C and the BP solution was slowly added dropwise to that of MEL. The mixture was then stirred continuously at 90 °C for 4 h so that MEL and BP fully reacted. After the reaction was complete, the product was washed to adjust the pH between 6.0 and 7.0, and the product was vacuum filtered, dried, and ground to obtain BPM as the final product. PM, FePM, and ZnPM were synthesized using the same methodology; however, in the absence of any metal precursor for PM, with ferrous acetate for FePM, and with zinc hydroxide for ZnPM.

### 2.3. Fabrication of Bimetallic Phytic Acid-Melamine Flame Retardant Coatings (BPME)

To prepare the BPM flame retardant coating, 3 g of BPM powder was dissolved in 20 mL of ethanol and stirred continuously for 30 min at 70 °C to obtain a homogeneous BPM/ethanol solution. Next, 25 g of EP was added to the solution and the mixture was stirred for 30 min. Finally, 1.52 g of EDA was added and the mixture was stirred for 5 min at room temperature to form a flame retardant BPME coating. The PM, FePM, and ZnPM flame retardant coatings were prepared using the same methodology and were named PME, FePME, and ZnPME, respectively.

### 2.4. Flame Retardant Coatings on Wood Surfaces (BPME-W)

BPME was coated onto the surface of wood to prepare flame retardant wood (BPME-W) for testing. The specific method involved coating one side of the wood surface with 0.3 mm of BPME with a brush and drying it for 24 h. After drying the same treatment is applied on the other side of the wood surface. The wood coated with pure EP, PME, FePME, and Zn-PME were named EP-W, PME-W, FePME-W, and ZnPME-W, respectively.

### 2.5. Characterization and Properties

#### 2.5.1. Structural Characterization

FTIR (Bruker, Saarbrücken, Germany) spectroscopy with a TENSOR27 instrument was used to analyze the chemical composition of the flame retardant powders. Small amounts of dried and KBr powders were taken separately, mixed well using an agate mortar, and subsequently made into thin slices for testing using a tablet press. The measurements entailed 32 scans and were performed within the wavenumber range of 400–4000 cm^−1^.

Powder X-ray diffraction (XRD) analysis was conducted using a Bruker AXS D8 Advance diffractometer to investigate the crystal structures of the flame retardants. The measurements were performed using the Cu target Kα line (λ = 0.15418 pm), with a step size of 0.01° and a scanning range of 5–80°.

The degree of graphitization of coating residue was analyzed via Raman spectroscopy using a HORIBA LabRAM HR Evolution Raman spectrometer (HORIBA Scientific, Paris, France) at a laser excitation wavelength of 532 nm.

The microscopic morphology, elemental distribution, and charcoal morphology of the flame retardants on the wood surface were analyzed by scanning electron microscopy (SEM; FEI, Hillsboro, OR, USA) and energy dispersive spectroscopy (EDS) with a Quanta 450 FEG field-emission scanning electron microscope (FEI, Hillsboro, OR, USA).

#### 2.5.2. Thermal Analysis

The thermal stability of the flame retardant coatings were investigated under a N_2_ atmosphere using an Is-50 thermogravimetric analyzer (Thermo Fisher Scientific, Waltham, MA, USA) with a carrier gas flow rate of 50 mL·min^−1^ and a protective gas flow rate of 20 mL·min^−1^. The test conditions ranged from 50 to 800 °C, and the rate of temperature increase was 10 °C·min^−1^.

#### 2.5.3. Flame Retardance and Burning Behavior

Flame retardance was tested according to the ASTM International Standard D2863 using a HC-2 Oxygen Index Meter (Jiangning Analytical Instrument Co., Nanjing, China) with the specimen size of 125.0 × 5.5 × 3.0 mm^3^. The samples were ignited using a top-plane ignition method to determine whether the burning time after ignition exceeded 300 s. If it did not, the oxygen concentration was increased, while if it exceeded 300 s, the oxygen concentration was lowered.

Based on the ASTM international standard D3801, flame retardance was tested using the CZF-3 vertical combustion tester (Jiangning Analytical Instrument Company, China) with a test specimen of 125.0 × 12.5 × 3.0 mm^3^. The height of the flame was adjusted to 2 cm, and the wood coated with the flame retardant was ignited twice for 10 s. The burning times of the material were recorded as t1 and t2, and the material was classified as V-rated based on the magnitude of t1 and t2. The combustion grades were categorized into V-0, V-1, and V-2, where V-0 is the highest grade, indicating that the composites displayed optimal flame retardant performance.

Based on the ASTM E1354/ISO 5660 standard, a TTech-GBT161172 cone calorimeter (Testech Technology, Suzhou, China) was used to test the heat release rate (HRR), smoke release, and other related parameters of the flame retardants on the wood surface during combustion. The size of the specimen was 100.0 × 100.0 × 3.0 mm^3^, the heat flux was 25.9 kw·m^−2^, and the separation distance was 25 mm. HRR represents the amount of heat released per unit of time from the combustion of a material, which is an important indicator for assessing the fire risk of a material, the peak heat release rate (PHRR) is the maximum value of HRR, and the total heat release (THR) is the total amount of heat released from the material throughout the combustion process. The time to ignition (TTI) represents the time interval between the exposure of a specimen to a heat source and the start of sustained combustion, whereas the smoke generation rate (SPR) and the total smoke production (TSP) during combustion are essential for assessing the effects of fire on human health. The CO_2_/CO ratio is the amount of carbon dioxide and monoxide produced during combustion, which aids in understanding the toxicity of the combustion products.

#### 2.5.4. Mechanical Properties

The hardness (PH), adhesion (AD), and glossiness (GS) of the coatings were characterized using the QHQ-A Pencil Hardness Tester (ASTM International Standard D3363-2005, Huaguo Precision Technology, Dongguan, China), the QHF hundred grid knife (ASTM International Standard D3359-09, Huaguo Precision Technology, Dongguan, China), and the HST-60A gloss tester (ASTM International Standard D523, Huiste, Shenzhen, China). The impact strength (IS) of the composites was tested using a simple beam impact tester (Shanghai Hesheng Instrument Technology Co., Ltd., Shanghai, China) with an impact velocity of 3.8 m·s^−1^ and an impact energy of 5 J. The final result for each specimen was the average of five valid measurements, and the specimen dimensions were 80 × 10 × 3 mm^3^.

## 3. Results and Discussion

### 3.1. Characterization of the Flame Retardants

**Figure 2:**
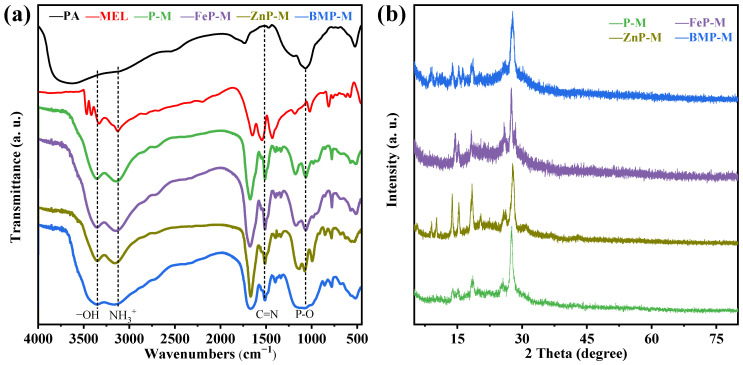
(**a**) FTIR and (**b**) XRD spectra of BPM flame retardant.

The FTIR spectra of flame retardant powders of PM, FePM, ZnPM, and BPM shown in Figure 2a provide evidence that the C–N telescoping vibration located at 782 cm^−1^ and the P–O stretch located at 1064 cm^−1^ for the flame retardant with single metal ion addition can be traced back to the presence of PA [18]. Moreover, the P–O bending vibration peak located at 522 cm^−1^ in PA shifted to 507 cm^−1^ in the FePM flame retardant (P–O telescoping vibration peak (P–O–Fe)) and to 534 cm^−1^ in ZnPM flame retardant (P–O telescoping vibration peak (P–O–Zn)), and appear in the composite flame retardant after combining phytic acid with metal ions [19]. However, in the flame retardant doped with Zn-Fe bimetallic ions, the position of the P–O (P–O–BM) telescopic vibrational peak was unchanged, suggesting that the Zn-Fe ions interact with each other, thus affecting the vibration of the P–O bond. The extent of the shift in wavenumbers indicated the involvement of phytic acid in chelation with metal ions. Sharp peaks at 3345 cm^−1^ (–NH^3+^ telescoping vibration) and 3120 cm^−1^ (–OH telescoping vibration) were observed for melamine in PM, FePM, ZnPM, and BPM [20]. Meanwhile, the C=N broadening vibration peak at 1508 cm^−1^ was attributed to the typical triazine ring of PM [15]. The XRD patterns of the flame retardant powders PA, MEL, PM, FePM, ZnPM, and BPM are shown in Figure 2b. In the range of 10–30°, all samples exhibited similar diffraction peaks, proving that the samples with added metal ions still had diffraction peaks corresponding to PM, which in turn indicated the successful introduction of metal ions into the flame retardant. The successful synthesis of NH_4_PO_2_(NH_2_)_2_ from phytic acid and melamine was evidenced by the presence of characteristic diffraction peaks at 2*θ* = 13.8, 14.9, 15.1, 18.5, 25.4, 27.8, and 37.2° (PDF#16-0915). Compared with PM, ZnPM showed new diffraction peaks at 2*θ* = 9.8 and 10.2°, which were attributed to the formation of the Zn_2_P_2_O_7_ hydrate (PDF#35-0071), and a diffraction peak at 31.2°, which was assigned to NH_4_Zn(PO_3_)_3_ (PDF#21-0745). Notably, the diffraction peaks of the ZnPM flame retardant in the range of 10–20° were sharper and narrower, indicating that the doping of zinc ions not only preserved the basic structure of PM but also improved the crystallinity of the flame retardant. Compared to ZnPM and PM, a new diffraction peak appeared at 28.5° for FePM, which was attributed to Fe_3_(PO_4_)_2_ (PDF#49-1087), directly proving the successful addition of Fe ions into the structure of PM. In addition, the diffraction peaks in the range of 10–20° also exhibited narrower peak widths, indicating that doping with iron ions similarly improved the crystallinity of the flame retardants. The diffraction peaks of BPM contained phases corresponding to PM, FePM, and ZnPM, which proved that the Zn and Fe bimetallic ion phytate-melamine flame retardants were successfully prepared.

**Figure 3:**
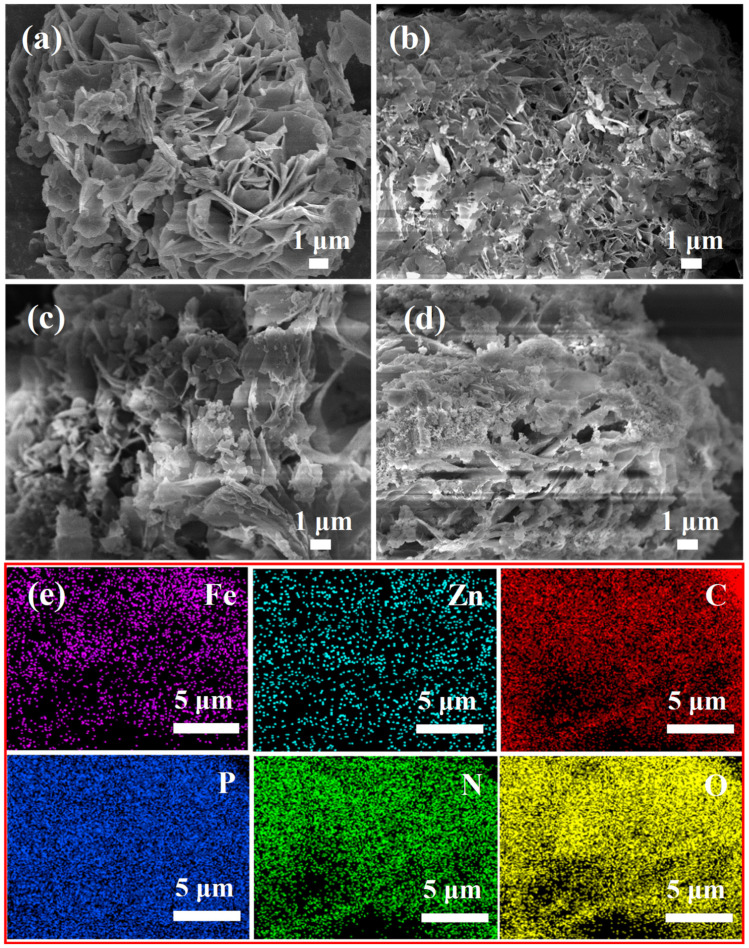
SEM images of (**a**) PM, (**b**) FePM, (**c**) ZnPM, and (**d**) BPM and (**e**) EDS elemental mapping of BPM.

The morphology of the flame retardants, as determined by SEM, is illustrated in Figure 3. PM, FePM, ZnPM, and BPM were stacked in fluffy nanosheets, which is conducive for dispersion in organic solvents or polymers. As shown in Figure 3b, white particles were observed on the surface of FePM, which may be due to the enrichment of Fe ions on the material surface or the formation of iron phosphates and other compounds. Figure 3c shows that the flaky structure of ZnPM is relatively rough, which may be due to Zn doping, which changes the crystallinity of the material and promotes crystal growth along specific orientations, consistent with the XRD results. Figure 3d shows that the layered structure of BPM was porous with the presence of a few particles on the surface. This dispersion may be due to the synergistic effect of Zn and Fe ions, which promote the dispersibility and stability of the layered structure of the material. The bimetallic ions may have formed new active sites between the layers or on the surface of the material, promoting delamination and uniform dispersion of the layered structure. Figure 3e presents the elemental mapping of the BPM nanosheets, which provides evidence that Zn, Fe, C, P, N, and O are evenly distributed. The uniform distribution of Zn and Fe, which are characteristic elements of BPM, further confirmed that Zn and Fe ions were successfully doped into the PM nanosheets.

### 3.2. Thermal Stability of the EP Composite Coatings

**Figure 4:**
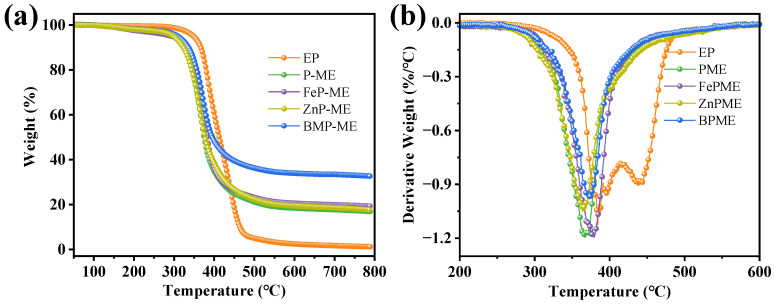
(**a**) TGA and (**b**) DTG curves of EP and EP composite coatings.

The thermal degradation behavior and thermal stability of the EP coating was characterized using thermal gravimetric analysis (TGA) in a nitrogen atmosphere. As shown in the mass-loss curves (Figure 4), the presence of fillers altered the decomposition mechanism of the EP matrix as the thermal degradation process of EP is divided into two steps. The addition of flame retardants resulted in the presence of a decomposition stage, likely due to the dehydration effect of the phosphorus-containing segments that accelerated the rate of degradation, thus forming a protective char [21]. The thermal stability curves and key parameters of the PME, FePME, ZnPME, and BPME flame retardant coatings are listed in Table 1. From Figure 4a, it is observed that the initial decomposition temperature (T_d5%_) of the EP composite material decreased to a varying extent after the addition of flame retardants. Similarly, the maximum degradation temperature (T_max_) of T_5%_ of PME, FePME, ZnPME, and BPME decreased to 299.9, 294.4, 298.3, and 309.0 °C, respectively, which corresponds to approximately a 13.9%, 15.5%, 14.4%, and 11.3% decrease, respectively. The peak temperature (T_max_) also decreased by approximately 4.3%, 0.9%, 4.7%, and 3.0%, respectively, owing to the catalytic effect of the phosphorus-containing degradation products of the flame retardants on the decomposition of the EP matrix [22]. Compared to pure EP, the char residue (CR) from PME, FePME, ZnPME, and BPME increased by approximately 12.0%, 13.8%, 12.5%, and 24.2%, respectively, and did not show significant changes after 500 °C, indicating that the flame retardants formed a protective char layer during the combustion process of the epoxy resin composite system, and that this char layer exhibited good thermal stability in the high-temperature region [23]. In particular, the presence of bimetallic ions in BPME resulted in the production of more residue. With the addition of flame retardants, the maximum rate of thermal decomposition increased, suggesting that the combination of phosphorus in phytic acid with Zn and Fe ions may form complexes with stronger catalytic effects. These complexes can effectively promote the carbonization and dehydration reactions of epoxy resins at high temperatures, thereby accelerating their decomposition rate [24]. Furthermore, the catalytic characteristics of Fe and Zn promoted the initial degradation of the composite material and catalyzed char production.

### 3.3. Flammability of the BPME-W Composites

Table 2 lists the parameters related to the LOI and UL-94 vertical burning rating. The EP-W exhibited an LOI value of 20.7%. Upon the addition of flame retardants PM, FePM, ZnPM, and BPM to the coating, the LOI value significantly increased by 16.4%, 31.4%, 27.5%, and 45.4%, respectively. These results indicate that the introduction of flame retardants, especially doping with Zn and Fe, greatly enhanced flame retardance of the epoxy resin on the wood surface through synergistic effects. Further analysis of the vertical burning test data (Table 2) revealed that the unmodified EP-W did not meet the UL-94 rating standards and exhibited dripping during combustion. This is due to the propensity for EP to undergo molecular chain scission during burning, forming smaller molecular fragments that are prone to dripping. While PME-W passed the UL-94 V-1 rating, significant improvements were observed in FePME-W, ZnPME-W, and BPME-W, all of which successfully passed the more stringent UL-94 V-0 rating test and effectively eliminated the dripping phenomenon observed during the combustion of EP-W. This suggests that the flame retardants form cross-linked structures with the matrix material upon exposure to high temperatures or open flame, effectively preventing the formation of small molecular fragments, thereby enhancing the material’s anti-dripping ability, which is crucial for preventing the spread of fire. Additionally, by testing the duration of the open flame after ignition, it was found that the epoxy resin coatings modified with metal-bio-based flame retardants exhibited shorter combustion times. Notably, the BPME-W sample showed an after-ignition time of only ~5–6 s, demonstrating superior self-extinguishing properties. This finding corroborates the results of the LOI test, together confirming the effectiveness of Zn and Fe doping in significantly enhancing the flame retardancy of bio-based flame retardants.

**Figure 5:**
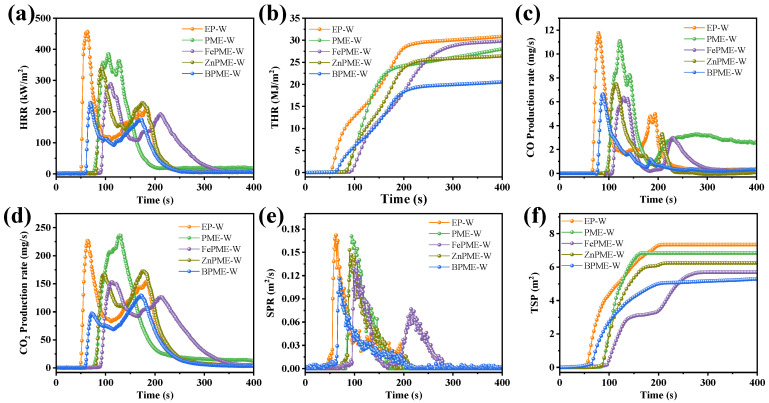
(**a**) HRR, (**b**) THR, (**c**) SPR, (**d**) CO production, (**e**) CO_2_ production, and (**f**) TSP curves of samples.

To further investigate the fire safety of the EP coating on wood with bimetallic flame retardants under actual combustion conditions, a cone calorimeter was used to test the HRR, THR, SPR, CO/CO_2_ production rate (PCO/PCO_2_), and TSP curves for EP-W, PME-W, FePME-W, ZnPME-W, and BPME-W; the results are presented in Figure 5 and Table 3. The TTI of EP-W was 54 s, indicating that it was highly combustible during fire. The incorporation of the flame retardants PM, FePM, ZnPM, and BPM enhanced the TTI of the EP on the wood surface. Specifically, the TTI values for PME-W, FePME-W, ZnPME-W, and BPME-W increased to 79, 89, 85, and 63 s, respectively. The increase in the TTI values is primarily attributed to the formation of a stable char layer by the flame retardants under high-temperature conditions, which acts as a barrier to flames and effectively delays the ignition of the material [25], indicating an improvement in ignition resistance. As shown in Figure 5a and Table 3, the EP-W curve exhibits a lower flame resistance and displays a pronounced peak (457.1 kW·m^−2^), whereas the formation of a stable protective carbon layer prevents further degradation of the EP matrix following the early stages of degradation for PME, FePME, ZnPME, and BPME, which is consistent with the TGA results. Consequently, the PHRR of the PME-W, FePME-W, ZnPME-W, and BPME-W modified flame retardants was reduced to 385.1, 291.7, 341.6, and 228.8 kW·m^−2^, respectively, representing a decrease of 15.8%, 36.2%, 25.2%, and 50.0%, respectively, as compared to EP-W. This reduction in PHRR demonstrates the effectiveness of the flame retardants in reducing the heat released during the combustion of EP. As the material surface continued to pyrolyze and char, an unstable carbon layer was formed, which slowed heat transfer to the interior of the material, thereby reducing the HRR. However, under continuous thermal radiation, the unstable carbon layer can be disrupted, allowing the internal material to pyrolyze and combust further, leading to the presence of a second peak in the HRR curve [26]. As shown in Figure 5b, the THR values for PME-W, FePME-W, ZnPME-W, and BPME-W were 17.7%, 2.9%, 13.8%, and 31.7% lower than that of EP-W, respectively. The HRR curves exhibit two peaks, which is a typical characteristic of IFRs [20]. This implies that the flame retardants played a significant role in the flame retardancy of EP on wood, with the incorporation of bimetallic ions notably reducing both PHRR and THR. CO is one of the most harmful gases produced during combustion and poses a substantial threat to human health. As illustrated in Figure 5c,d, PCO decreased with the addition of the flame retardants. Compared with EP-W (12 mg·s^−1^), the best-performing samples, FePME-W and BPME-W, both achieved a PCO reduction of 41.7% to 7 mg·s^−1^. Furthermore, PCO_2_ for EP-W was 229 mg·s^−1^, whereas that for FePME-W, ZnPME-W, and BPME-W exhibited decreases of 31.9%, 23.4%, and 42.4%, respectively. However, PME-W exhibited an increase of 3.8% in PCO_2_, which may be attributed to the catalytic effect of PM promoting the conversion of CO to CO_2_. The SPR and TSP curves of the EP and its composites are shown in Figure 5e,f. With the addition of flame retardants, both the peak smoke production rate (PSPR) and TSP decreased, with BPME-W showing the lowest values among all samples, reducing PSPR by 32.9%; EP-W produced a significant amount of smoke (7.2 m^2^). In contrast, the TSP of PME-W was reduced by 5.7%. The smoke emission results for FePME-W (5.7 m^2^), ZnPME-W (6.1 m^2^), and BPME-W (5.1 m^2^) indicate that the addition of metal ions can reduce the generation of smoke particles, and that the synergistic effect of bimetallic ions enhances their smoke suppression performance. The FGI parameter (defined as the value of the PHRR/tPHRR ratio) is an important parameter for evaluating flame retardant performance in fire protection technology [27]. A smaller FGI value indicates slower fire spread, thereby reducing the fire hazard of flammable polymers. As listed in Table 3, the FGI of EP-W was 7.26 kW·m^−2^s^−1^. In contrast, BPME-W showed the lowest FGI, with a 60% reduction, indicating that BPME-W demonstrated the lowest fire hazard among the samples. Therefore, melamine phytate doped with Zn and Fe ions can effectively suppress the release of heat, toxic gases, and smoke from EP composites, thereby significantly improving their flame retardant performance.

### 3.4. Char Residue Analysis

**Figure 6:**
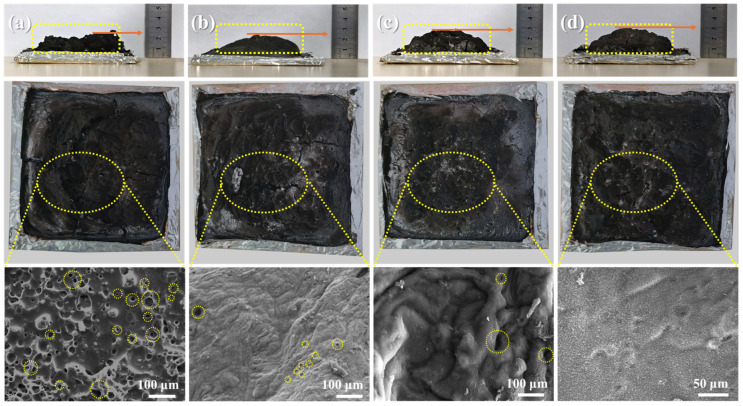
Digital and SEM images of char residues collected from CCT for (**a**) PME-W, (**b**) FePME-W, (**c**) ZnPME-W, and (**d**) BPME-W samples.

A cone calorimeter was employed to assess the process of char formation for the EP matrices with varying metal ions incorporated, whereas a detailed investigation of the morphology and composition of the char residues was conducted using SEM. These analyses aimed to elucidate the specific influence of Zn and Fe ions on the process of char formation of the EP matrix. Figure 6 displays digital photographs and SEM images of the char residues from PME-W, FePME-W, ZnPME-W, and BPME-W, evidencing a significant difference in char morphology compared to the original EP-W without added flame retardants [28]. As depicted in Figure S1, following the cone calorimeter test, the pristine EP-W had nearly entirely combusted and the remaining char was fragmented and porous, which was not conducive to forming an effective insulating barrier. The inability to form a barrier hinders the effective prevention of the transfer of combustible volatiles and heat from the condensed phase to the flame zone. In the case of PME-W (Figure 6a), the char thickened and increased in mass; however, the surface still exhibited numerous pores, indicating that at high temperatures, phytic acid melamine decomposes to form phosphorus-containing acidic substances, which can catalyze the formation of a char layer to some extent. For FePME-W (Figure 6b) and ZnPME-W (Figure 6c), the char surface possessed a reduced number of small pores, appearing dense and intact overall. This indicates that the incorporation of metal ions improves the structure of the char layer, thereby enhancing its smoke suppression properties. Notably, the char layer of ZnPME-W was thicker than that of FePME-W, indicating that Zn ions played a more significant role in the char formation process (consistent with the TGA results). As shown in Figure 6d, the synergistic effect of the Zn and Fe bimetallic ions in BPME-W further enhanced the compactness and integrity of the char layer, which was more conducive to suppressing heat release and smoke production. This synergistic effect may be due to the different mechanisms by which the various metal ions contribute to char formation and flame retardation, including free radical scavenging, catalyzing char formation, and improving the physical structure of the char layer.

**Figure 7:**
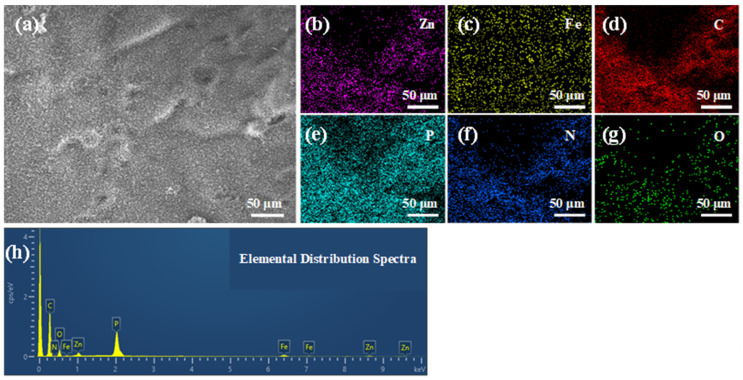
SEM images (**a**), EDS elemental mapping (**b**–**g**), and ((**h**), Table 4) elemental occupancies of char residues for the BPME-W sample.

The elemental distribution on the surface of the char residue for BPME-W revealed the presence of Zn, Fe, C, P, N, and O, which all exhibited a uniform distribution across the surface of the char layer (Figure 7b–h). Table 4 lists the elemental composition of the char residue surface, with a C content of 72.22 wt.% and P content reaching 7.41%. This indicates that PM predominantly acts within the condensed phase, retarding the decomposition of the EP matrix. The nitrogen content was only 3.70 wt.%, which can be attributed to esterification reactions that form numerous cross-linked structures during combustion [29], with the majority of nitrogen being released in the form of NH_3_ [13,30]. The residue contained 3.70 wt.% Fe and 1.86 wt.% Zn, further confirming that Fe and Zn remained in the condensed phase to catalyze the dehydrogenation and aromatization of the intermediate products generated by the polymer during combustion, thereby enhancing the integrity of the protective char layer [31].

**Figure 8:**
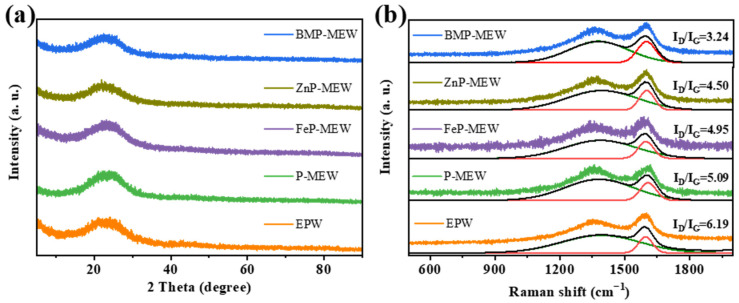
(**a**) XRD and (**b**) Raman of char residues collected from CCT for all samples.

As shown in Figure 8, further XRD and Raman spectroscopic analyses of the char residue formed after the cone calorimeter test of the EP composite material were conducted to obtain detailed structural information. Figure 8a shows the presence of a broad diffraction peak in the range of 20–35° for the post-combustion materials, indicating that the composite materials possessed typical amorphous characteristics; that is, the synthesized materials were amorphous.

The degree of graphitization, which provides information regarding the quality of the char residue after combustion, was examined using Raman spectroscopy (Figure 8b). Two characteristic Raman shifted peaks were observed in all curves: the D-band at 1360 cm^−1^, which corresponds to the presence of amorphous carbon structures; and the G-band at 1580 cm^−1^, whose intensity is closely related to the graphite content [32]. The relative ratio of I_D_ to I_G_, where I_D_ and I_G_ are the integrated area ratios of the D-band and G-band, respectively, indicates the degree of graphitization of the char [33]. A small relative ratio corresponds to a high degree of graphitization, indicating better char quality and stronger flame retardance [34]. The results evidenced that when different additives (PM, FePM, ZnPM, and BPM) were added to the EP matrix, the relative ratio of I_D_ to I_G_ for the composite materials decreased from 6.19 to 3.24. The addition of BPM significantly promoted graphitization of the carbonized products, as indicated by the lower I_D_/I_G_ ratio. The char yield data in Table 1 further confirm the enhancing effect of BPM on the char-forming ability of the EP-based composite material. Therefore, the highly graphitized characteristics of the residual char from the bimetallic ion composite material during the combustion process may be a key factor in the formation of an effective condensed phase barrier on the wood surface. This barrier not only slows down heat transfer and oxygen permeation, but also significantly improves the overall flame retardance of the material through a physical barrier mechanism.

**Figure 9:**
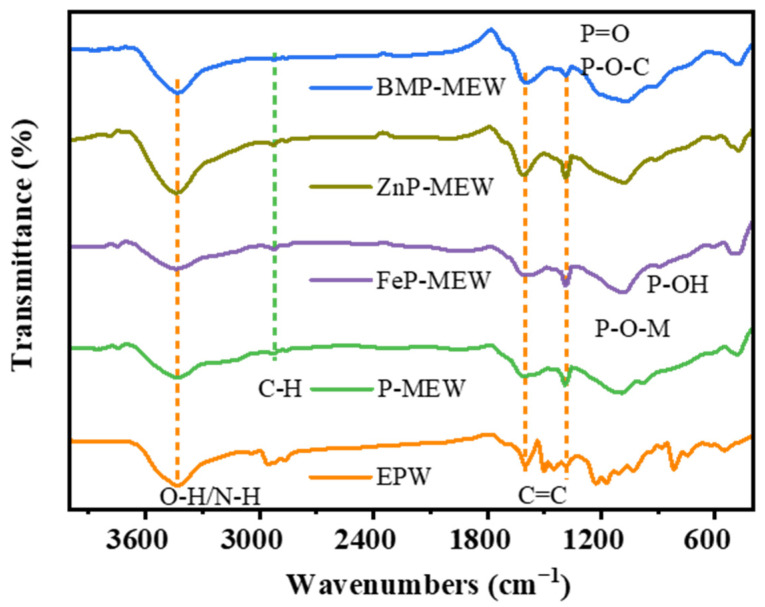
Raman spectra of char residues collected from CCT for all samples.

The Raman spectra contain a characteristic absorption peak at 3440 cm^−1^, which is attributed to the O–H/N–H stretch [35], whereas characteristic peaks for P=O and P–O–C bonds appeared at 1139 and 1160 cm^−1^ [36], respectively, suggesting that decomposition can produce polyphosphoric, metaphosphoric, or pyrophosphoric acids (Figure 9). Polyphosphoric acids can undergo esterification reactions with the EP substrate, forming organic char with a P–O–C structure, while metal oxides formed from the decomposition of metal ions can further create a metallic protective layer. This continuous and dense dual-effect protective layer can block heat transfer and prevent the spreading of flames [37]. The C–H stretching vibration band at 2920 cm^−1^ [38], the characteristic peak for a C=C stretch at 1600 cm^−1^ [39], and the high transmittance observed between 882–899 cm^−1^ (P–N, P–OH) [40] indicate that BPM is able to generate phosphoric acid during pyrolysis, thus promoting esterification and dehydration reactions of the EP matrix and thereby facilitating the formation of a phosphorus-containing aromatic char layer. The coverage of metal oxides on the surface of the char layer further enhanced the structure and thermal stability of the EP char layer. Moreover, owing to the synergistic catalytic effect of Zn and Fe ions, the formation of various carbon products within the char layer structure was promoted. This reduced the release of heat and smoke during combustion, protecting the polymer matrix and preventing further decomposition [41], thus increasing the flame retardance of the EP composites.

### 3.5. Flame Retardant Mechanism

**Figure 10:**
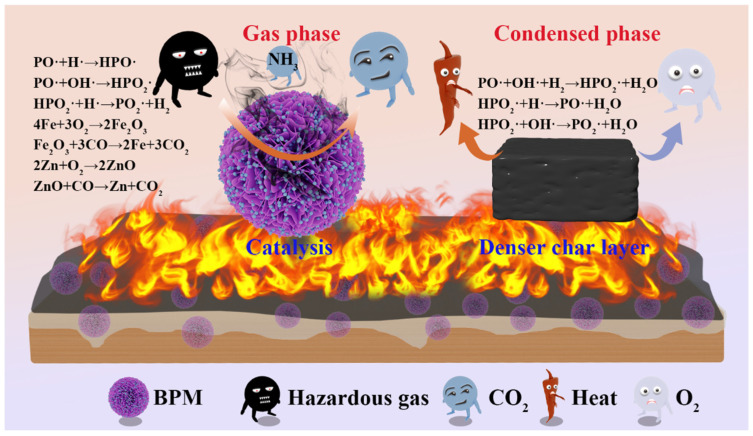
Schematic illustration of flame retardant mechanism for EP and EP composite coating on wood surface.

Considering the evolution of the successive roles of BPM in suppressing EP fire hazards, the mode and mechanism of fire protection in the gas and condensed phases were investigated separately (Figure 10). PA, MEL, and metal ions contribute together to the formation of a stable cross-linked network that promotes the development of an expanded coke layer on the surface of EP materials.

In the gas phase, free radical quenching effects and dilution effects are mainly involved. PA Phosphorus radicals (PO·, HPO_2_·) generated during decomposition capture H· and OH· generated during combustion [42], thus interrupting the chain reaction of combustion. Nitrogen-containing compounds in melamine decompose at high temperatures, releasing large amounts of nonflammable gases such as N_2_ and CO_2_ [43]. These gases dilute the concentration of combustible gases and oxygen in the air, thus reducing the rate and intensity of combustion; some metastable metal ions can be used as free radical traps or oxidation catalysts to inhibit the production of smoke and harmful gases [44]. The condensed phase mechanism mainly involves a physical barrier effect, catalytic carbonization, and catalytic cross-linking. Firstly, the decomposition of PM produces phosphate-containing acids, such as phosphoric acid and pyrophosphoric acid, which promote the dehydration and carbonization of the epoxy resin chains to form a dense carbon layer [45]. Then, bimetallic Zn-Fe ions in BPM decompose into metal oxides that promote the formation of internally expanded carbon and act synergistically in catalytic carbonization at higher temperatures, resulting in a higher carbon residue [46]. At the same time, the lamellar structure forms tortuous “porous paths” within the polymer matrix that can act as physical barriers, lengthening the path of volatiles and heat transfer and reducing the rate of combustion. Finally, metals are reduced in situ to metal oxides, which facilitates CO reduction.

In summary, the flame retardants in EP synergize their flame retardant effects in both the gas and condensed phases, significantly improving the thermal stability and flame retardance of the material, thus providing enhanced protection.

### 3.6. Mechanical Performance

**Figure 11:**
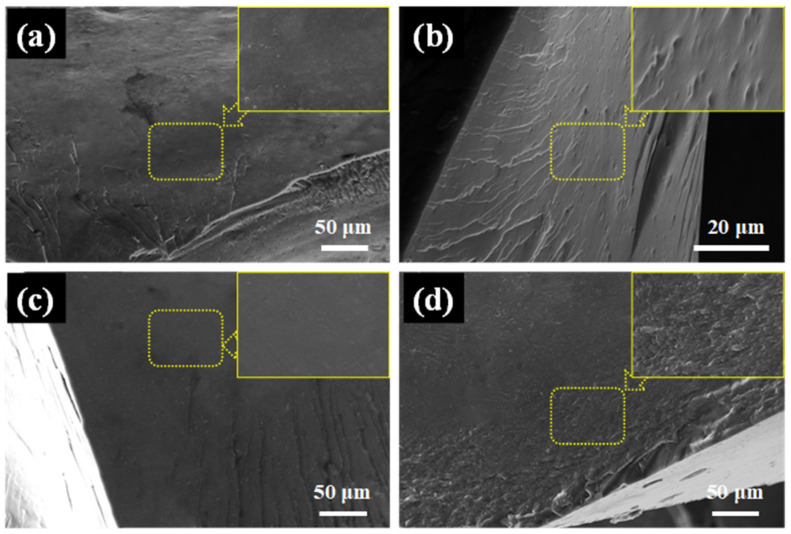
The fracture morphology of (**a**) PME, (**b**) ZnPME, (**c**) FePME, and (**d**) BPME after impact testing.

The compatibility of the EP with the added flame retardants directly affects the mechanical properties and flame retardance of the EP nanocomposite coatings. The fracture surface of the composite coatings was analyzed using SEM to demonstrate the compatibility of the flame retardant with the epoxy matrix. As shown in Figure S2, the fracture surface of pure EP was smooth, reflecting its brittleness and weak resistance to crack propagation [47]. As shown in Figure 11, the fracture surface of PME exhibits a wrinkled and microcracked structure. With the addition of FePM, ZnPM, and BPM, the fracture surfaces became rougher, indicating increased energy dissipation and toughness. This phenomenon can be attributed to the increased presence of intermolecular forces such as hydrogen bonds, which enhance the toughness of the material [48]. Generally, the binding energy of a system with hydrogen bonds increases significantly, leading to the absorption of more energy during tensile or impact loading, which improves the mechanical properties [49]. In addition, no agglomeration of nanoparticles was observed on the fracture surface. It has been reported that the active –NH_2_ groups in the flame retardant can react with epoxy groups at high temperatures, thereby improving the compatibility between the EP matrix and the flame retardant [50].

**Figure 12:**
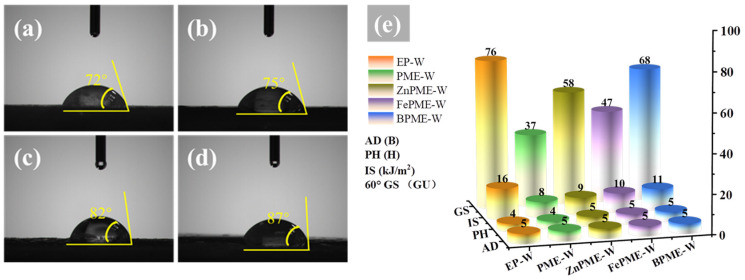
Photographs illustrating the contact angle obtained for (**a**) PME-W, (**b**) FePME-W, (**c**) ZnPME-W, and (**d**) BPME-W. (**e**) Mechanical properties of all samples.

From an industrial application perspective, it is crucial to retain the mechanical properties of EP on wood surfaces while improving flame retardance. Therefore, the water contact angle (WCA), IS, GS, AD, and PH of the EP composite coatings on wood surfaces were studied. Water resistance is an important criterion for assessing service lifetimes of materials in humid environments. In this study, the hydrophobicity of coated wood surfaces was evaluated using WCA measurements. As shown in Figure S3, the WCA of the EP-W was 69°, indicating that its surface was hydrophilic. The hydrophilicity of EP is mainly due to the presence of a large number of hydroxyl groups on the surface after curing, which readily form hydrogen bonds with water molecules, thereby limiting its application in environments with high humidity. Figure 12a shows that the WCA of the EP modified with PM on the wood surface was 72°. This small increase may be attributed to the incorporation of PM, which led to changes in the chemical structure and spatial arrangement of the hydroxyl groups on the EP surface, thereby improving its hydrophobicity. The phosphoric acid groups in PM may react with the hydroxyl groups in EP, reducing the density of the surface hydroxyl groups, thus lowering the surface energy and increasing the hydrophobicity. The WCA of FePME-W, ZnPME-W, and BPME-W increased to 75°, 82°, and 87°, respectively, indicating that the doping of metal ions not only improves the hydrophobicity but also enhances the interfacial performance of the coating by forming new chemical bonds or altering the surface roughness. Doping with metal ions may promote the formation of a more compact and uniform network structure on the coating surface, which helps impede the penetration of water molecules, thereby enhancing the waterproofing performance of the coating. The IS of EP-W reached 16 kJ·m^−2^; however, that of PME-W, FePME-W, ZnPME-W, and BPME-W decreased by 50%, 38%, 44%, and 38%, respectively. This may be due to the poor interfacial compatibility between the flame retardant and EP, as well as the addition of metal ions that restrict the mobility of the EP molecular chains, causing the material to become more rigid and reducing its ability to deform and absorb energy upon impact [51]. As shown in Figure 12e, the GS of EP-W was 76, while that of PME-W, FePME-W, ZnPME-W, and BPME-W decreased, possibly because of the addition of flame retardants, which increased the roughness of the resin surface or modified the refractive index and scattering characteristics of the resin. The flame retardant particles may also cause surface irregularities, thereby reducing specular reflection, increasing light scattering, and lowering glossiness. There was no significant change in AD, which may be due to the fact that the added flame retardants enhanced the chemical bonding or mechanical interlocking between the resin and wood substrate. For instance, Zn and Fe ions may react with the hydroxyl groups in wood, or their particles may form more micro-anchors on the wood surface, thereby improving adhesion. Compared to the pencil hardness of 4H for EP-W and PME-W, that of FePME-W, ZnPME-W, and BPME-W increased to 5H, which may be due to the addition of flame retardants, which increased the cross-linking density of the resin, thereby enhancing its hardness and scratch resistance.

## 4. Conclusions

In this study, we successfully developed a novel flame retardant composed of Zn-Fe double metal salts of phytic acid and melamine, and applied it to EP coatings to enhance the flame retardance of wood. The flame retardant coating on BPME formed an efficient flame retardant layer on the wood surface, significantly improving its flame retardant performance. Specifically, in TGA, LOI tests, and cone calorimetry experiments, the char yield and LOI value of BPME increased by approximately 24.2% and 45.4% as compared to EP, respectively. Meanwhile, it significantly reduced the HRR and THR during wood combustion, with a 50.0% reduction in PHRR and a THR value that was 31.7% lower than that of EP-W. Additionally, BPME-W demonstrated excellent smoke suppression performance, with a 41.7% reduction in PCO values, a 29.2% decrease in TSP, and a 60% reduction in FGI as compared to EP-W, indicating its significant benefits in terms of minimizing fire hazard risk. These results indicate that MEL complexed with the bimetallic salts of PA can effectively suppress the release of heat, toxic gases, and smoke from EP composites. This study further explored the synergistic flame retardant mechanism between the Zn-Fe double metal salts of PA and MEL and found that their combined action promoted the carbonization process on the wood surface, forming a denser char layer that effectively blocked the spread of oxygen and heat. This discovery therefore provides a new theoretical foundation and practical guidance for the application of EP coatings for flame retardance on wood.

## Figures and Tables

**Table 1:** Thermal data of EP and its composites under N_2_ atmosphere.

Samples	T_d5%_ (°C)	T_max_ (°C)	CR (wt.%)
EP	348.4	383.8	1.3
PME	299.9	367.2	16.9
FePME	294.4	380.2	17.6
ZnPME	298.3	365.8	19.3
BPME	309.0	372.3	32.7

**Table 2:** LOI values and UL-94 ratings of EP and its composites on a wood surface.

Samples	LOI (%)	t1 (s)	t2 (s)	Dripping	Cotton Ignition	Rating
EP-W	20.7	>60	-	Yes	Yes	No
PME-W	24.1	12.8	19.1	No	No	V-1
FePME-W	27.2	7.9	15.9	No	No	V-0
ZnPME-W	26.4	8.2	9.8	No	No	V-0
BPME-W	30.1	1.5	5.6	No	No	V-0

**Table 3:** Cone calorimetry data for different specimens.

Sample	TTI (s)	T_PHRR_ (s)	pHRR (kW·m^−2^)	THR (MJ·m^−2^)	P_CO_ (mg·s^−1^)	P_CO2_ (mg·s^−1^)	PSPR (m^2^·s^−1^)	TSP (m^2^)	FGI (kW m^−2^·s^−1^)
EP-W	54	63	457.1	30.16	12	229	0.173	7.2	7.26
PME-W	79	107	385.1	24.8	11	238	0.171	6.8	3.60
FePME-W	89	88	291.7	29.3	6	156	0.139	5.7	3.31
ZnPME-W	85	93	341.6	26.01	8	175	0.153	6.1	3.67
BPME-W	63	79	228.8	20.6	7	132	0.116	5.1	2.90

**Table 4:** Elemental Distribution Spectra.

Element	Wt.%
C	72.22
N	3.70
O	11.11
P	7.41
Fe	3.70
Zn	1.86

## Data Availability

The original contributions presented in the study are included in the article/Supplementary Material, further inquiries can be directed to the corresponding authors.

## Supplementary Materials

The following supporting information can be downloaded at: https://www.mdpi.com/article/10.3390/polym16243586/s1, Figure S1: The char residues of EPW from CCT was studied by digital and SEM images; Figure S2: The fracture morphology of EP after impact testing; Figure S3: The contact angle of EP-W.
