# Supplementary material for: Synthesis of a Bimetallic-Doped Phytate-Melamine Composite as an Efficient Additive for Epoxy Resins with High Fire Safety
Source: Polymers (Basel). 2024 Dec 21;16(24):3586. doi: 10.3390/polym16243586 (PMC11679077; doi:10.3390/polym16243586)
Supplement: Supplementary file 1 [file polymers-16-03586-s001.zip › polymers-3356884-supplementary.pdf]

Supplementary Information

Synthesis of bimetallic-doped phytate-melamine composite as an efficient additive for high fire safety epoxy resin

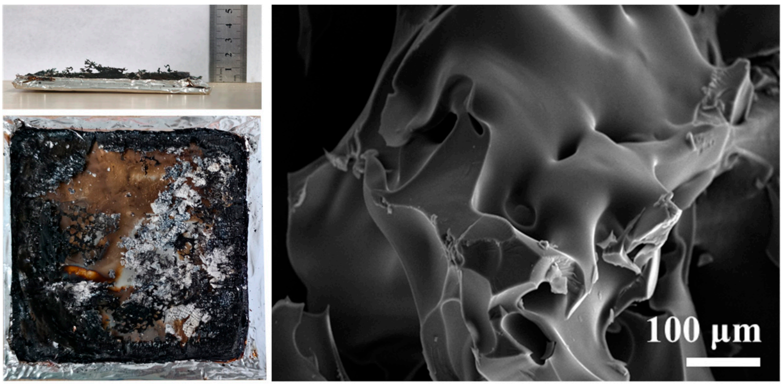

Fig. S1 The char residues of EPW from CCT was studied by digital and SEM images.

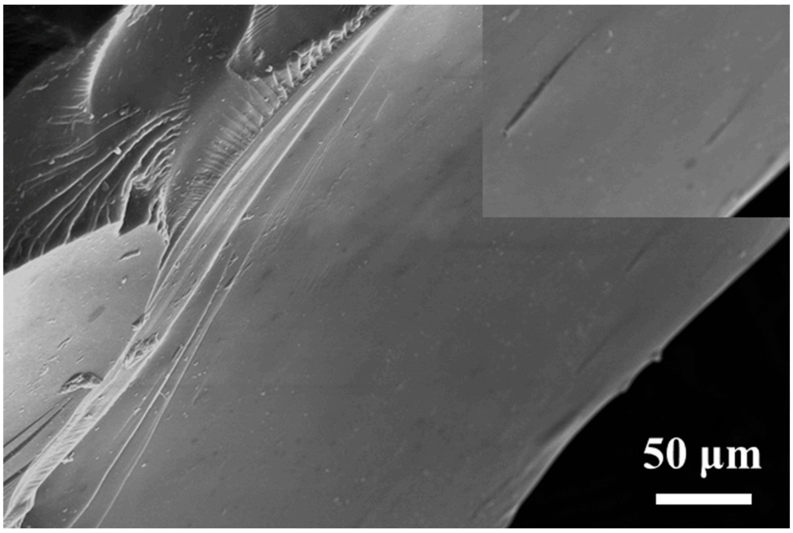

Fig. S2. The fracture morphology of EP after impact testing.

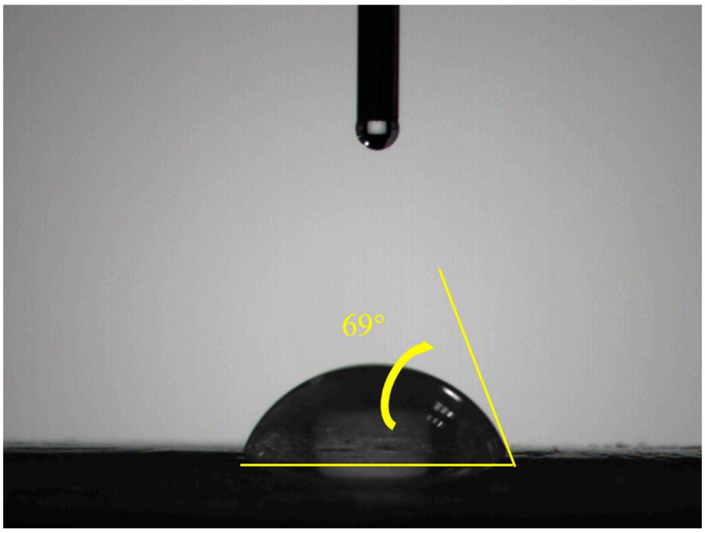

Fig. S3. The contact angle of EP-W.
